# The PRECISE-DYAD protocol: linking maternal and infant health trajectories in sub-Saharan Africa

**DOI:** 10.12688/wellcomeopenres.18465.2

**Published:** 2024-04-05

**Authors:** Rachel Craik, Marie-Laure Volvert, Angela Koech, Hawanatu Jah, Kelly Pickerill, Amina Abubakar, Umberto D’Alessandro, Benjamin Barratt, Hannah Blencowe, Jeffrey N Bone, Jaya Chandna, Melissa J. Gladstone, Asma Khalil, Larry Li, Laura A Magee, Liberty Makacha, Hiten D Mistry, Sophie E. Moore, Anna Roca, Tatiana T Salisbury, Marleen Temmerman, Danielle Toudup, Marianne Vidler, Peter von Dadelszen, and The PRECISE-DYAD Network

**Affiliations:** 1 Department of Women and Children’s Health, School of Life Course and Population Sciences, Faculty of Life Sciences and Medicine, King’s College London, London, UK; 2 Nuffield Department of Women's and Reproductive Health, University of Oxford, Oxford, UK; 3 Centre of Excellence Women and Child Health, Aga Khan University, Nairobi, Kenya; 4 Medical Research Council Unit The Gambia at the London School of Hygiene and Tropical Medicine, Fajara, The Gambia; 5 Department of Obstetrics and Gynaecology, University of British Columbia, Vancouver, Canada; 6 Institute for Human Development, Aga Khan University, Nairobi, Kenya; 7 MRC Centre for Environment and Health, Imperial College London, London, UK; 8 London School of Hygiene and Tropical Medicine, London, UK; 9 Institute of Life Course and Medical Sciences, University of Liverpool, Liverpool, UK; 10 Fetal Medicine Unit, Department of Obstetrics and Gynaecology, St. George's University Hospitals NHS Foundation Trust, London, UK; 11 Vascular Biology Research Centre, Molecular and Clinical Sciences Research Institute, St George's University of London, London, UK; 12 Department of Surveying and Geomatics, Midlands State University, Gweru, Zimbabwe; 13 Health Service and Population Research Department, Institute of Psychiatry, Psychology and Neuroscience, King’s College London, London, UK; 14 Medical School, University of Sheffield, Sheffield, UK

**Keywords:** Maternal health, child health, neurodevelopment, global health, pregnancy complications, biorepository, air quality

## Abstract

**Background:**

PRECISE-DYAD is an observational cohort study of mother-child dyads running in urban and rural communities in The Gambia and Kenya. The cohort is being followed for two years and includes uncomplicated pregnancies and those that suffered pregnancy hypertension, fetal growth restriction, preterm birth, and/or stillbirth.

**Methods:**

The PRECISE-DYAD study will follow up ~4200 women and their children recruited into the original PRECISE study. The study will add to the detailed pregnancy information and samples in PRECISE, collecting additional biological samples and clinical information on both the maternal and child health.

Women will be asked about both their and their child’s health, their diets as well as undertaking a basic cardiology assessment. Using a case-control approach, some mothers will be asked about their mental health, their experiences of care during labour in the healthcare facility. In a sub-group, data on financial expenditure during antenatal, intrapartum, and postnatal periods will also be collected. Child development will be assessed using a range of tools, including neurodevelopment assessments, and evaluating their home environment and quality of life. In the event developmental milestones are not met, additional assessments to assess vision and their risk of autism spectrum disorders will be conducted. Finally, a personal environmental exposure model for the full cohort will be created based on air and water quality data, combined with geographical, demographic, and behavioural variables.

**Conclusions:**

The PRECISE-DYAD study will provide a greater epidemiological and mechanistic understanding of health and disease pathways in two sub-Saharan African countries, following healthy and complicated pregnancies. We are seeking additional funding to maintain this cohort and to gain an understanding of the effects of pregnancies outcome on longer-term health trajectories in mothers and their children.

## Background

Worldwide, there are 46,000 maternal and 2.5 million fetal, neonatal, and infant deaths per year associated with pregnancy hypertension, fetal growth restriction (FGR) and stillbirth ^1^, and more than half of them occur in sub-Saharan Africa ^1^. The interplay between maternal health, common pregnancy complications, and subsequent maternal and child health trajectories has been described in high income countries ^2– 7^, including the SCOPE study [ https://www.scopestudy.net/]. Although studies have demonstrated that preterm birth, infections in pregnancy and hypoxic-ischaemic encephalopathy are linked to poorer child health and neurodevelopmental delay, the mechanisms by which pregnancy complications may precede or be related is not clear and has not been well studied – particularly in low- and middle-income settings and in Africa, where the risks for delay are more common ^8– 10^. 

Women and children living in sub-Saharan Africa (SSA) face multiple social and health challenges that have negative impacts on pregnancy outcomes and may predispose women to common disorders of pregnancy. Such challenges include: calorie-restricted and variety-limited diets; chronic and acute infections; exposure to poor indoor and exterior air quality ^11, 12^, lack of clean water and sanitation; extreme weather events (e.g. extreme heat, floods and droughts), limited decision-making regarding health and social care; and, challenging geographical access to health care facilities ^13, 14^. In SSA, women with pregnancy complications have higher rates of related morbidity and mortality than in high-income countries, yet very little is known of the longer-term health consequences following placental disorders.

PRECISE-DYAD seeks to address this area of neglected global health research. It will expand and extend the PRECISE study (PREgnancy Care Integrating Translational Science, Everywhere [ PRECISEnetwork.org]), which is a broadly-based group of research scientists and health advocates, mainly based in Africa and the UK. PRECISE is established a network through a shared project that investigates three important complications of pregnancy, namely high blood pressure (hypertension), babies who are smaller than they should be before birth (fetal growth restriction) and babies who die before birth (stillbirth). PRECISE has a deeply-phenotyped maternal-fetal/newborn pairs to provide insights into the life course of health and disease ^15^. The main novelty of this study is this is one of the first detailed pregnancy and child follow-up biorepositories covering East, West and Southern sub-Saharan Africa and includes both data as well as biological samples, alongside measures of child and neurological developmental measures. The focus of PRECISE-DYAD is to understand the resilience and vulnerability pathways leading to varied maternal and child health outcomes, following either healthy pregnancies or those with complications, namely pregnancy hypertension, preterm birth, FGR and stillbirth.

## Aim and objectives of PRECISE-DYAD

To test the overarching hypothesis that maternal-fetal-child health trajectories in sub-Saharan Africa are inextricably linked through social, clinical, and biomarker factors, the broad aim of PRECISE-DYAD is to provide an epidemiological and mechanistic understanding of maternal and infant health and development, in relation to in utero disease pathways in sub-Saharan African settings. The specific objectives are:

1) To assess the effect of pregnancy complications on mothers (i) decisions around birth spacing (ii) subsequent pregnancy (iii) mental health (iv) child bonding (v) early-onset non-communicable disease (NCD) and multi-morbidities, and (vi) social functioning.
2) To assess the measurable impact of pregnancy events on physical, mental, and neurodevelopmental health trajectories (specifically, moderate-to-severe neurodevelopmental, visual, or hearing disability) of children at 2–3 years of age.
3) To (i) identify the biological mechanisms that underpin relationships between intrauterine and early postnatal exposures, e.g. environmental or infectious diseases and maternal and child outcomes and (ii) investigate if health trajectories are modified by environmental exposures (e.g., air and water quality) and co-exposures (e.g., nutrition, lifestyle and quality of care) during the postnatal period and (iii) assess what the effects of caring for children with moderate-to-severe neurodevelopmental disability (vs those without such disability) are on: (a) subsequent pregnancy, (b) maternal mental health, and (c) early-onset NCDs and multi-morbidities.

## Protocol

### Study design

**Figure 1.**
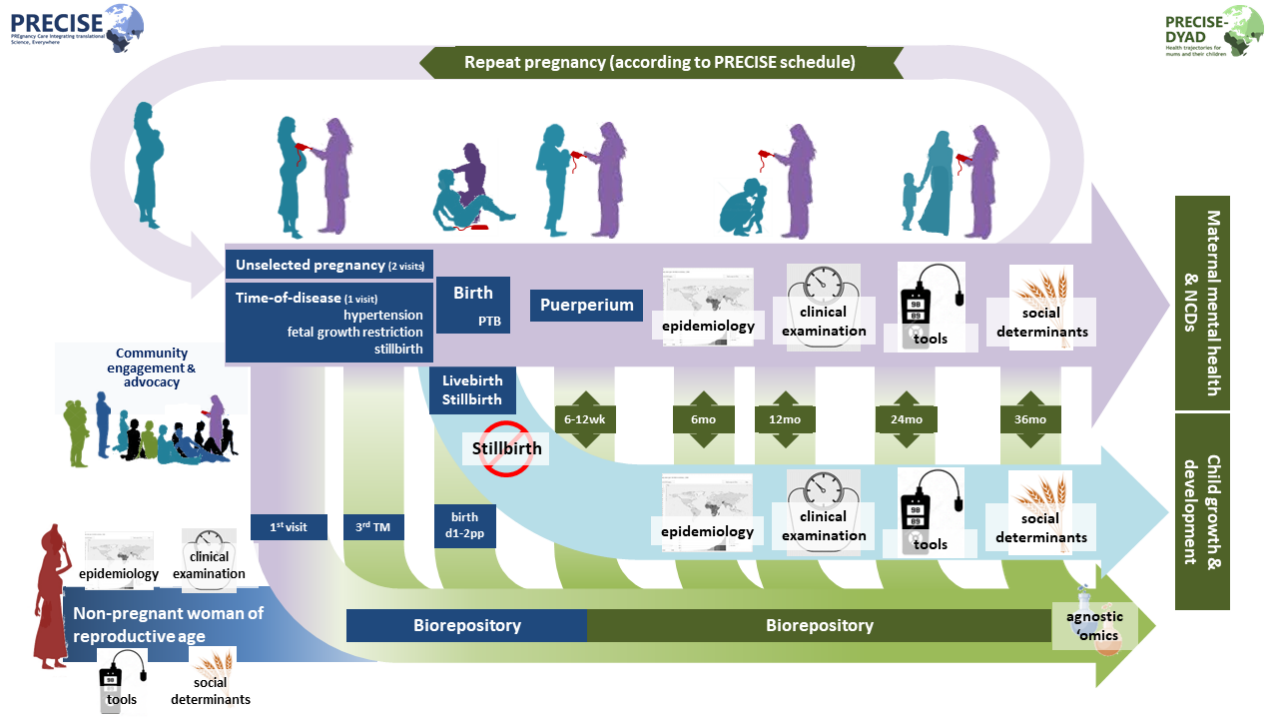
Schematic of cohort activities: PRECISE (blue) & PRECISE-DYAD (green). **NCD**, Non-Communicable Disease; **PTB**, Preterm Birth; **pp**, PostPartum; **TM**, Trimester.

PRECISE-DYAD is an observational study of a cohort of women and their children in The Gambia and Kenya. The participants were previously recruited into the PRECISE study ^15– 17^ and we are now going to follow them up for two years. Building upon the detailed pregnancy information and samples established by PRECISE ^15– 17^, PRECISE-DYAD will collect additional biological samples and further clinical data on both the maternal and child health, including their general health, medications, nutrition, hospital admissions, the child’s neurodevelopment and maternal mental health and physical health, including clinical assessments to measure blood pressure, pulse oximetry and anthropometry measurements ( Figure 1). We will continue to take a holistic approach, considering social determinants of physical and mental health, alongside a biorepository that complements replete clinical and epidemiological data.

### Research settings

**Figure 2.**
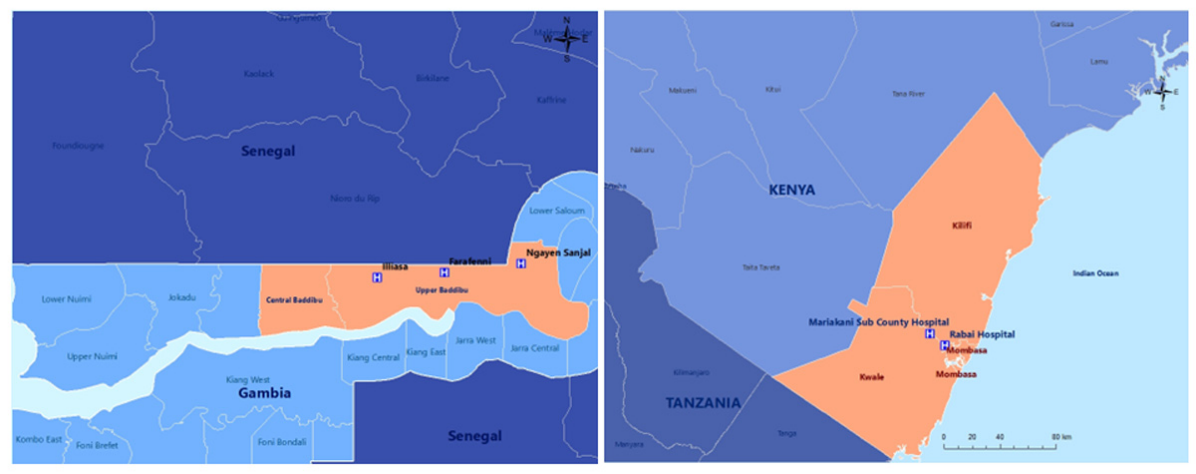
Sites in The Gambia (left) and Kenya (right).

***The Gambia.*** Our primary partner in The Gambia is the MRC Unit The Gambia at the London School of Hygiene and Tropical Medicine (MRCG at LSHTM). Field research will take place at the Maternal Newborn Child and Adolescent Health clinic in Farafenni (urban primary health centre (PHC)), the Farafenni General Hospital, and associated rural PHCs in Illiasa and Ngayen Sanjal ( Figure 2 left).

***Kenya.*** Our primary partner in Kenya is the Aga Khan University. The field research will be conducted in urban Mariakani Subcounty Hospital and rural Rabai Subcounty Hospital ( Figure 2 right).

### Community engagement

Extensive community engagement has been undertaken since 2018 as part of the PRECISE study by the research teams working closely with the participating communities in Kenya and The Gambia ( Table 1). As we transition into the PRECISE-DYAD study, the new study and its protocols will be integrated into these sessions to mitigate any concerns prior to the study commencing. We will continue to focus on building strong relationships to ensure the research study is understood and the research team are mindful and sensitive to cultural or religious beliefs, particularly on the collection of biological samples.

**Table 1.** Community engagement.

	The Gambia	Kenya
**Stakeholders**	Former PRECISE participants Health care workers Community Birth Companions Village Health Nurses Community leaders, traditional and religious	Government County Ministry of Health Health care workers Community leaders Community health volunteers Community-wide meetings Pregnant women (former PRECISE participants), their partners, family members including children
**Engagement ** **methods**	Community Engagement event within the community or at the community’s health facility Local radio announcement PRECISE-DYAD open days	Health talks (baraza) Community meetings Meetings for target audiences (pregnant women, mothers, in-laws, partners) PRECISE-DYAD open days
**Key messages**	Information and interactive sessions on maternal and child health with aspects of NCD, maternal nutrition, child nutrition, WASH, biological samples, maternal mental health, child development, and COVID	Introduction to research and informed consent Information sessions on maternal mental health, nutrition, child development and healthy environments When/how/why of biobanking
**Engagement ** **frequency**	Continuation of PRECISE sensitisation activities Sensitisation activities occur continuously before and during individual follow-up; Radio sensitisation occurs monthly Community Engagement events occur twice monthly Open Days are held twice a month	Continuation of PRECISE activities with increased focus in areas with the largest numbers of participants Open days are held twice a month whilst there are about 8 community meetings and one health worker meeting each month.

In addition, we will be hosting ‘PRECISE-DYAD open days’ in the participating communities. These will involve interactive learning activities and stimulated discussions about pregnancy and maternal and child health. Open days will provide the opportunity to learn about the women’s experiences of being involved in the study as well as provide the community/participants with the opportunity to bring to our attention any emerging issues related to the study. Through these events, we hope to increase participants’ understanding of the PRECISE-DYAD study and to create a general awareness of research and its potential benefit to the communities. Each participant, their families and community members will be invited to the open days and the activities have been planned to ensure they do not impact on the study itself particularly around the outcomes of child development.

A brief overview of planned community activity is presented in Table 1.

### Consent process

Women participating in PRECISE will be invited to be a part of PRECISE-DYAD. They will be given the information sheet and consent form, either written or verbally, and consent will be confirmed with the participant’s signature or a thumbprint. In cases of a thumbprint, a literate witness (other than the member of the research team obtaining consent) will be asked to sign the consent form as well. 

### Confidentiality

All consenting and interviews will take place in the local health facilities in an appropriate space for privacy. Hard copies of study-related forms will be stored in a locked cabinet in a storage room under supervision of the principal investigators as per policy of the hosting institution. Electronic records will be stored in password-protected computers and tablets and only approved study personnel will have access to the entire set of information. All specimens and associated phenotypic data will be de-identified and given a unique participant identifier and no personal information will be stored in the specimen tracking data management system.

## Data and sample collection

Mothers and/or infants will be followed-up for two years; with contact points at: 6 weeks to 6 months, 12 months, 24 months, and 36 months (if the child reaches this age within the 2 years of data collection from July 2021 to July 2023). Any maternal or child deaths will be investigated by performing a verbal autopsy at a culturally acceptable time within the data collection period. We will use the World Health Organization (WHO) Verbal Autopsy tool which is designed for all age groups, including maternal and perinatal deaths. Through a standardised set of questions including the person's general health, symptoms, medication use and the lead up to the death, probable causes of death will be derived ^18^. An overview of the visit content for women is described in Table 2 and for children in Table 3.

**Table 2.** Data collection and sampling from mothers.

Mother Assessment	Visit 1 (6 wk-6 months after birth)	Visit 2 (12 months after birth)	Visit 3 (24 months after birth)	Visit 4 (36 months after birth)
**General information**	●	●	●	●
**Medication**	●	●	-	-
**General Health**	●	●	-	-
**COVID-19**	●	●	●	●
**Environment + WASH** (if women have moved homes)	●	●	●	●
**Contraception** (if women are not pregnant)	●	●	●	●
**Pregnancy intention** (if women are pregnant)	●	●	●	●
**Nutrition**	●	●	●	●
**Pelvic Floor**	●	-	●	-
**Quality of Care** (nested case control study)	●	-	-	-
**Mental health** (sub-study of 300 women/site)	●	●	-	-
**Health economics** (case control sub-study)	●	-	-	-
**Vital signs** *(BP, HR, Pulse Ox, Haem, RR, Peak flow)*				
**Anthropometry** *(height, weight, MUAC, waist: hip * *circumference)*	●	●	●	●
**Cardiology Assessment** *(pulse wave and cardiac * *output)*	-	●	-	-
**Biological sample collection**	●	●	●	●

**Table 3.** Data collection and sampling from children.

Children assessment	Visit 1 (6 wk-6 months after birth)	Visit 2 (12 months after birth)	Visit 3 (24 months after birth)	Visit 4 (36 months after birth)
**General health** *(includes vaccination questions)*	●	●	●	●
**Nutrition**	●	●	●	●
**Neurodevelopment**	●	●	●	●
**Breathing question**	-	●	●	●
**Vital signs** ( *BP, HR, Pulse Ox, Haem, RR*)		●	●	●
**Anthropometry** *(height, weight, head * *circumference, MUAC)*	●	●	●	●
**Biological sample collection**	●	●	●	●

**General data principles:** Where possible, all study visits will be held in the study facilities to enable full data collection. Phone interviews will be conducted, when necessary, in Kenya if the participant is unable to attend the health facility. Given the extent of the data collection, appointments are expected to take 2–3 hours. The study team will ensure that women and their children are comfortable and provided with refreshments. All the clinical data will be captured directly onto the database, ODK-X ^19^, using Android tablets. As this is an observational study, any individuals with suspected health issues will be referred into the existing health system pathway for appropriate care. 

Data related to the air quality, and water, sanitation, and hygiene (WASH) components will be collected at the participant’s home. In Kenya, the health economics sub-study will also be conducted at home.

**Non-clinical data:** At each PRECISE-DYAD visit, we will collect a standard set of non-clinical data relating to both the mother and child including their home environment and family structure. If they have moved homes since the last study visit, we will ask additional questions about their natural and built environment (e.g., water source and housing materials).

**Clinical data:** Women will be asked about both their and their child’s health since their previous study visit as well as information on their diets. All participants are being invited to participate, via phone calls and once signed consent has been obtained at their first visit, are invited back for up to 4 visits, where different detailed clinical questions, neurodevelopmental and cardiological assessments and biological samples are collected at the various timepoints as detailed within the protocol manuscript. We will collect anthropometric and vital sign measurements from both mother and child, including height, weight, mid upper arm circumference (MUAC) as well as blood pressure, respiratory rate, pulse oximetry, haemoglobin, and peak respiratory flow measures. Due to the COVID-19 pandemic, we will ask questions related to COVID-19 symptoms and capture information about COVID-19 vaccinations. For the mothers, we will conduct a basic cardiology assessment one year after the woman delivered using two devices, the arteriograph ^20^ to measure arterial stiffness and central pulse wave reflection, and the Ultrasonic Cardiac Output Monitor (USCOM) ^21^ to measure cardiac output and systemic vascular resistance. These data will be used to assess possible risk factors of cardiac disease.

**Neurodevelopmental assessment:** Child development will be assessed using a range of tools at each visit. The Malawi Developmental Assessment Tool (MDAT) will be used in all children to assess for neurodevelopmental outcomes, with age specific questions being asked at each visit ^22^. In addition, we will observe the quality of interactions between the mother-child dyads or, in the event of an unavailable mother, between the child and their primary caregiver, using the Observation for Maternal-Child Interaction tool (OMCI) ^23^. For the children that are between 12 and 16 weeks of age, we will assess their spontaneous movement using the Prechtl’s Assessment of General Movements to predict later cerebral palsy ^24, 25^. Throughout the study, we will screen for epilepsy, learn about their home environment through the Family Care Indicators questionnaire ^26^, use the Neurodevelopment Screen Tool ^27^ to screen for neurodevelopmental disorders and the Pediatric Quality of Life Inventory (PedSQL) ^28^ to assess their quality of life. For children where concerns are flagged as developmental milestones are not being met, additional assessments will be carried out, including the Cardiff test to assess vision and the Modified Checklist for Autism in Toddlers to evaluate for the risk of autism spectrum disorder (ASD) ^29^. Any child who is not meeting the expected milestones will be referred for follow-up through the standard clinical care pathway.

**Mental health**: As this is a sub-study to look at the feasibility of asking women in these settings about their mental health, mental health will be assessed in the first 300 women in each country to attend their first PRECISE-DYAD visit 6 weeks – 6 months after delivering. For each woman we will use four validated measures; for depression (Patient Health Questionnaire; PHQ-9) ^30, 31^, anxiety (Generalised Anxiety Disorder; GAD-7) ^31– 33^, post-traumatic stress disorder (Post-traumatic Stress Disorder Checklist – civilian version; PCL-C) ^34^, and functional impairment (World Health Organization Disability Assessment Schedule; WHODAS 2.0) ^35^. All tools will be adapted and translated into local languages where necessary and piloted prior to use to ensure utility and comprehension. Women will be assessed twice, at PRECISE-DYAD visit 1 (6 weeks – 6 months postpartum) and again at PRECISE-DYAD visit 2 (12 months postpartum), except for WHODAS which is done at visit 1 only.

**Quality of care:** This nested case-control study will include 300 cases and 300 controls, where women will be asked (via interviews at the appropriate visit), about their experiences of care during labour and while giving birth in the healthcare facility. Women will be selected as cases based on the mode of delivery and outcome: caesarean deliveries, perinatal deaths, cases of severe hypertension - systolic BP >160mmHg and/or diastolic BP >110mmHg, and babies born small for gestational age <3rd percentile or preterm <33 weeks. Women with uncomplicated pregnancies and term, normal weight live births will be taken as controls. The data collected will help to understand the role of the quality of care in the PRECISE-DYAD facilities to provide insights into women’s experiences in the health system.

**Air quality:** Personal air quality will be assessed using portable sensor packs (Dyson Technology Ltd, Malmesbury, UK) carried by the women for 5 days, which continuously monitor PM _2.5_, PM _10_, nitrogen dioxide, temperature, humidity, and mobility (accelerometry and GPS location). Measurements are logged every second autonomously for 120 hours. The air quality study will also be assessed in Mozambique in a subset of women who participated in the PRECISE study, as part of the PRECISE-HOME study in two health facilities Manhiça District Hospital and Xinavane Rural Hospital. A cohort of 50 women per health facility in all three countries will be recruited based on their geographic location and fuel use to ensure the sample is representative of the study population. Three study sites in The Gambia, two in Kenya and two in Mozambique result in 350 households, each of them with two measurements, one in the dry season and the other in the rainy season. These devices are not very heavy and fit in a small shoulder bag and following some community engagement, it was found that mothers were happy to where these. Using the solar-powered Clarity Node-S low-cost sensors, additional fixed sampling to assess ambient air quality will be carried out continuously outside each recruitment centre (n=7) during the wet and dry seasons (~8 months).

**WASH:** We will assess water quality in the same households involved in the air quality study (i.e., 250 households). A water sample will be taken from the water source and another sample from the water the mother drinks. In the Gambia this will be done at two time points, once in the dry season and once in the rainy season. In Kenya the water sampling will only be done once. A water sample of 1000ml from the source and 500ml of drinking water will be collected in sterile containers. In the source water we will look at physical-chemical analytes (chloride, nitrate, sodium, calcium, magnesium, total iron, sulphate, manganese, copper, phosphorus, potassium, zinc, total alkalinity, total hardness, conductivity, pH, residual chlorine, total dissolved solids), and heavy metals (lead, arsenic, and mercury). Furthermore, on both water samples we will conduct microbiological tests for bacterial contamination looking at the coliform count and *Escherichia coli*. This will be linked to an additional questionnaire to capture data on water, sanitation, and hygiene in the home. These data will be used with the air quality data, combined with geographical, demographic, and behavioural variables to create personal environmental exposure models for the full cohort.

**Health economics:** This case-control sub-study will involve 100 participants per country, comprising of 50 cases selected using the same criteria for the quality of care sub-study described above and 50 controls. The study aims to determine whether the occurrence of a pregnancy complication affects a family’s opportunity costs (e.g., direct heath care expenditure, loss of earnings) and economic resilience (e.g., food security), during and after pregnancy. Women will be interviewed at home or at the health facility to collect data on financial expenditure during antenatal, intrapartum, and postnatal periods.

**Pregnancies in PRECISE-DYAD:** Capturing the data on new conceptions taking place during PRECISE-DYAD will greatly enrich the dataset as the outcome of these new pregnancies can be compared with that of former pregnancies. If a participant is pregnant, we will ask for her informed consent to participate in the study and be followed up throughout her pregnancy in a similar process as in the PRECISE study. We hope this data will give a better understanding on the effect of pregnancy complications on decisions around birth spacing as well as the effect of caring for children with moderate-to-severe neurodevelopmental disability (vs those without such disability) on subsequent pregnancies.

For women who become pregnant during the PRECISE-DYAD study, we will conduct two study visits during pregnancy and a third at delivery. At each visit we will apply a sub-set of the original PRECISE questionnaire, conduct a non-invasive clinical assessment as familiar to the participant from the PRECISE study (i.e., taking blood pressure, heart rate, measure haemoglobin, body weight, and collect maternal blood, maternal urine, cord blood and placental samples. At delivery we will collect a few drops of blood from the heel of the newborn. Should the pregnant participant develop a placental complication such as hypertension in pregnancy, suspected fetal growth restriction or stillbirth, an additional study visit would be conducted at the time of admission to hospital.

**Biorepository**: Several biological samples will be collected from the mother and child throughout the study ( Table 4). All samples described below will be collected, processed, and stored in adherence to the PRECISE-DYAD Network Biological standard operating procedures (SOP). All laboratory staff have been trained through the PRECISE study to ensure they understand how to process samples.

**Table 4.** Samples collected in PRECISE-DYAD.

Samples	Visit 1 (6 wk-6 months after birth)	Visit 2 (12 months after birth)	Visit 3 (24 months after birth)	Visit 4 (36 months after birth)	Total Collections
**Maternal ** **blood**	16 ml	16 ml	16 ml	16 ml	4
**Breast milk**	5 ml				1
**Maternal ** **vaginal swab**	4 swabs				1
**Maternal ** **Urine**		20 ml	20ml		2
**Child Blood**	2-3 drops (heel prick)	2-3 drops (finger prick) or 5ml venepuncture	2-3 drops (finger prick) or 5ml venepuncture	2-3 drops (finger prick) or 5ml venepuncture	4
**Child Stool**	2 swabs		2 swabs		2

The sample collection of PRECISE-DYAD will grow the valuable PRECISE biorepository, creating an extensive biorepository with the potential to unravel mechanisms and identify novel biomarkers (with/without clinical data) as predictors of important maternal and child adverse outcomes.

### Study sample size

For sample size calculations, we have estimated that we will achieve up to 80% follow-up for three years after birth ^36– 39^. This will result in a cohort of ≈4200 women and ≈3950 children (assuming 6% stillbirths and neonatal/infant deaths). Of the related pregnancies, approximately 960 will have been complicated by a placental disorder ^40, 41^.

### Analytical approach

A variety of statistical approaches will be applied depending on the specific domain of interest. Descriptive analyses of data will be computed as appropriate depending on variable type. For inferential analyses relating to the effect pregnancy exposures of interest on maternal and child outcomes, we will use methods of causal inference for observational data such as inverse probability weighting or g-methods ^42^. Mediation analyses will be used to further disentangle paths between exposures and maternal and child outcomes, where possible mediators can be identified ^43^. In all analyses we will include confounders and mediators based on expert input and a conceptual framework informed by literature. For sub-cohorts (e.g., mental health) where women were matched during sampling, analyses will account for matching via generalized estimating equations or mixed effects models. All effect estimates will be accompanied by appropriate 95% confidence intervals. Missing data will be handled by multiple imputation by chained equations with a minimum of 50 imputed data sets. To assess the impact of unmeasured variables, or possible selection bias, quantitative bias analyses or bounding (e.g., E-values) may be considered ^44, 45^. 

## Co-ordination

Overall co-ordination of PRECISE-DYAD will be led by the central team at King’s College London (KCL) in collaboration with the team at University of British Columbia (UBC). Both the Kenyan and the Gambian sites will have their own research team consisting of co-PIs, clinical leads, data managers, laboratory managers, nurses and fieldworkers who will coordinate and implement the study locally. 

## Data management

The data collection platform used for PRECISE-DYAD will be the same as for PRECISE. For clinical data, we will use the electronic data capture system ODK-X. Each country will host their own database on secure servers and will be managed by local IT teams. Data will be collected on tablets and will be synced daily to the central database. To ensure high quality of data, program rules will be integrated to implement skip logics, and cross validation rules will be created for checking inconsistencies. In addition, the sites will run their own query reports weekly to clean the data and the central team at KCL will run monthly query reports.

The laboratory information management system (LIMS) used for this study, as for PRECISE, will be OpenSpecimen ^46^. The database supports secure online data entry on a standard web browser, or offline data collection on a mobile device and data synchronisation with the server over Wi-Fi connection by end users. As with ODK-X, programming rules will be implemented to minimise data entry errors, and query functions will be programmed in the database so these can be run by the team at any time to resolve issues.

## Ethics approval and consent to participate

Approval for the PRECISE-DYAD study was obtained from King’s College London (Ref HR-20/21-19714), The Gambia Government/MRC Joint Ethics Committee (Ref 22843), Aga Khan University, Nairobi Institutional Ethics Review Committee (Ref 2021/IERC-08) and University of British Columbia (Ref H20-02769).

Approval for the PRECISE-HOME study was obtained in King’s College London (Ref HR-17/18–7855), the Mozambique Ministry of Health, National Bioethics Committee for Health (CIBS-CISM/105/2021).

## Study status

The PRECISE-DYAD study started clinical activity in July 2021, where children 1 year and under were invited to participate in the study (visits 1 and 2). In October 2021, some children turned 2 years old so the 3rd visits commenced, with the 4th visits starting in October 2022. To date, we have recruited 768 participants in The Gambia and 1469 in Kenya.

## Discussion

This will be a unique cohort of mother-child dyads that includes both healthy and complicated pregnancies in health facilities in urban and rural communities in two sub-Saharan African countries. The mother-child dyads will provide data with which to compare the context of women and their children, their environment, biology, and outcomes. These culturally and geographically relevant data will aid identifying pathways to health resilience or vulnerability as pregnant women’s burden of infectious, mental, and non-communicable diseases will be considered, thus addressing the current research gap in global health. There is also the potential to introduce novel methods to assist the prevention, diagnosis, and management of the sequelae of placental disorders in sub-Saharan Africa, through for example new diagnostics based on the agnostic ‘omic screening of samples from women and children following complicated and uncomplicated pregnancies. Our aim is to conduct intervention and implementation trials that are responsive to what is learnt in the PRECISE-DYAD study.

Implementing this study will raise several challenges that we have tried to mitigate as much as possible. The study visit length of 2–3 hours will be challenging for mothers and especially for young children. We will provide refreshments and breaks during the period participants are in the health facility. We have tried to find a balance between the frequency and length of the visits to try and minimise the number of participants losing interest or those feeling overburdened by the study. As this is expected to be a long-term follow up study, participant fatigue and potential loss to follow up or withdrawal may occur. To try and mitigate this, all sites have established community engagement activities consisting of information and feedback sessions so that community members always have opportunities to ask questions or raise concerns and remain engaged in the study. We also used these activities to have in-depth discussions on the best approach within a given community for the collection of samples that may have cultural or religious significance such as maternal and child blood or breast milk samples. Participants are also made aware that they have the right to withdraw from the study at any time with no consequence. We also plan to conduct qualitative studies to gain understanding of the communities’ perceptions surrounding the research, for example, on biobanking.

We are seeking additional funding to continue the follow-up of this cohort of women and children, to gain an understanding of the effects of healthy and complicated pregnancies on longer-term health trajectories in mothers and their children. 

We will use the PRECISE-DYAD open days to deliver findings relevant and important to the participating communities. To communicate with health authorities, scientific researchers, advocates, and funders outside of our current network, we have developed as part of The PRECISE Network a comprehensive communication strategy. Through collaborating and sharing resources with governments, multilaterals and NGOs, PRECISE-DYAD will provide the means to plan and implement interventions and protective measures to support healthy pregnancies and child development as well as tackle the negative effects of complicated pregnancies and unfavourable health, social and environmental conditions experienced by mothers and their children to ultimately reduce maternal and child morbidity and mortality. 

## Abbreviations

ASD: Autism spectrum disorder; BP, Blood pressure, HR, heart rate, Pulse Ox: pulse oximetry, Haem: Haemoglobin, RR: respiratory rate, PEDSQL: Pediatric Quality of Life DDS: dietary diversity score; FGR: Fetal growth restriction; KCL: King’s College London; LSHTM: London School of Hygiene and Tropical Medicine; LIMS: Laboratory Information Management System; MDAT: Malawi Developmental Assessment Tool; MRC: Medical Research Council; MRCG at LSHTM: MRC Unit The Gambia at the London School of Hygiene and Tropical Medicine; MUAC: Mid upper arm circumference; NCD: Non-communicable disease; PHC: Primary health centre; PRECISE: Pregnancy Care Integrating translational Science, Everywhere; PTB: Pre-term birth; SOP: Standard operating procedure; UBC, University of British Columbia; USCOM: Ultrasonic cardiac output monitor; WASH: Water, sanitation and hygiene; WHO: World health organization.

## Definitions

Hypertension in pregnancy: This is defined as a clinic systolic BP ≥ 140 mmHg and/or a diastolic BP ≥ 90 mmHg, with systolic BP ≥ 160 mmHg and/or a diastolic BP ≥ 110 mmHg defined as severe hypertension ^47^.

Gestational hypertension: This is defined as hypertension arising *de novo* at ≥ 20 weeks’ gestation in the absence of proteinuria or other findings suggestive of preeclampsia ^47^

Preeclampsia ( *de novo*): This is defined as gestational hypertension accompanied by one or more of the following new-onset conditions at ≥ 20 weeks’ gestation:

i) Proteinuria
ii) Other maternal end-organ dysfunction, including neurological complications (e.g., eclampsia, altered mental status, blindness, stroke, clonus, severe headache, or persistent visual scotomata), pulmonary oedema, haematological complications (e.g., platelets <150,000/microlitre, disseminated intravascular coagulation, haemolysis), acute kidney injury (e.g., creatinine >90 µmol/litre or >1mg/dL), liver involvement (e.g., elevated transaminases with or without right upper quadrant or epigastric abdominal pain)
iii) Uteroplacental dysfunction (e.g., placental abruptio, angiogenic imbalance foetal growth restriction or intrauterine fetal death) ^47^

Stillbirth: This is defined as an **infant** born with no signs of life after a given threshold, usually related to the gestational age or weight of the baby; in this study we will use both the current World Health Organization (WHO) definition for international comparison of a stillbirth as being ‘a baby born without signs of life at or after 28 weeks of gestation’ ^48^ and the more inclusive definition of birth of an **infant** without signs of life ≥500g or ≥20 ^0^ weeks of gestation ^49^).

Preterm birth: The WHO defines preterm birth as any birth before 37 completed weeks of gestation (fewer than 259 days since the first day of the women’s last menstrual period) ^50^.

Small for gestational age: This is defined as **infants** (ex utero) weighing less than the 10 ^th^ centile birth weight for gestational age and sex. We will use the multi-ethnic, INTERGROWTH-21 ^st^ birth weight standard ^41, 51^.

Fetal growth restriction: This refers to a fetus (in utero) that has failed to reach its biological growth potential ^41, 51^

## Data Availability

No data are associated with this article
