# Supplementary material for: The PRECISE-DYAD protocol: linking maternal and infant health trajectories in sub-Saharan Africa
Source: Wellcome Open Res. 2024 Apr 5;7:281. Originally published 2022 Nov 14. [Version 2] doi: 10.12688/wellcomeopenres.18465.2 (PMC11109552; doi:10.12688/wellcomeopenres.18465.2)
Supplement: Peer review history

# Reviewer report: Reviewer response for version 2

**DOI:** 10.21956/wellcomeopenres.23499.r79218

**Published:** 2024-05-07

**Authors:** Billie F Bradford

**Affiliations:** 1 Obstetrics and Gynaecology, Monash University, Clayton, Victoria, Australia

Thank you for the revisions and excellent work. I have no further comments.

Is the study design appropriate for the research question?

Yes

Is the rationale for, and objectives of, the study clearly described?

Yes

Are sufficient details of the methods provided to allow replication by others?

Yes

Are the datasets clearly presented in a useable and accessible format?

Not applicable

Reviewer Expertise:

Pregnancy, maternity care, and perinatal mortality.

I confirm that I have read this submission and believe that I have an appropriate level of expertise to confirm that it is of an acceptable scientific standard.

## References

[1] : The PRECISE-DYAD protocol: linking maternal and infant health trajectories in sub-Saharan Africa. *Wellcome Open Research* .2024;7: 10.12688/wellcomeopenres.18465.2 10.12688/wellcomeopenres.18465.2 PMC1110955238779418

# Reviewer report: Reviewer response for version 2

**DOI:** 10.21956/wellcomeopenres.23499.r79219

**Published:** 2024-04-15

**Authors:** Tao Xue

**Affiliations:** 1 Peking University, Beijing, Beijing, China

Thank the authors for answering my questions in details. I have no further comments.

Is the study design appropriate for the research question?

Yes

Is the rationale for, and objectives of, the study clearly described?

Yes

Are sufficient details of the methods provided to allow replication by others?

Yes

Are the datasets clearly presented in a useable and accessible format?

Yes

Reviewer Expertise:

Reproductive epidemiology; Environmental epidemiology.

I confirm that I have read this submission and believe that I have an appropriate level of expertise to confirm that it is of an acceptable scientific standard.

# Reviewer report: Reviewer response for version 1

**DOI:** 10.21956/wellcomeopenres.20478.r65523

**Published:** 2023-09-27

**Authors:** Billie F Bradford

**Affiliations:** 1 Obstetrics and Gynaecology, Monash University, Clayton, Victoria, Australia

The authors have already established the PRECISE pregnancy cohort biorepository in sub-saharan Africa and now present a protocol for a set of follow-up sub-studies among PRECISE mothers and their offspring (including subsequent pregnancies) in The Gambia and Kenya.

 The proposed collections of clinical and non-clinical data, social, health, and environmental information seek to explore linkages between pregnancy and perinatal outcomes and social and environmental factors. As such, this proposal makes use of the resource established by the PRECISE network including biosample repository and trained personnel, facilities etc.

 The proposed set of sub-studies constitute an ambitious programme of work with some associated risk. Some of the data collection requirements may be demanding for the study population, risking insufficient recruitment or retention of participants. The protocol would benefit from more detail on data collection instruments and methods to provide reassurance about the feasibility.

 Further information on the following would be helpful;

1. A brief summary of PRECISE in the introduction
2. Under the Clinical data heading it states that women will be asked about both their and their child's health. How will they be asked? Will this be via survey or interview? How many language groups are included amongst the PRECISE-DYAD population and how will this be overcome?
3. The Mental Health study is referred to as a pilot study, but may be better described as a sub-study that will include a pilot phase.
4. The quality of care sub-study would also benefit from detail on how data will be collected from women on their care experiences (survey/interview?).
5. The Air Quality sub-study will require participants (mothers presumably) to carry a device for 5 days. Further detail shroud be provided on the potential burden to participants and how this might be offset to ensure recruitment and retention.
6. Details on data collection instruments are also scant in the Health Economics sub-study.

Finally, the study sample size consideration relates to placental disorders among the PRECISE-DYAD population and so it remains to be seen whether the group sizes of the substudy participants are sufficient in regards to the endpoints.

 There is some redundancy in the writing and further detail could be added on the specific methods for each sub-study without making the article excessively long.

 Despite the comments above, the group undertaking this work have already demonstrated the ability to carry out ambitious work in this context. Perinatal outcomes are considerably worse in sub-saharan Africa than in other parts of the world representing a greater burden for families and a downstream brake on social and economic advancement. Thus, there is an imperative to conduct research that may inform understandings of factors which promote healthy pregnancy and birth outcomes and those which contribute to harm. The PRECISE network have established the means to conduct such studies and have the skills and experience to do so, despite the challenges I have highlighted.

Is the study design appropriate for the research question?

Yes

Is the rationale for, and objectives of, the study clearly described?

Yes

Are sufficient details of the methods provided to allow replication by others?

Yes

Are the datasets clearly presented in a useable and accessible format?

Not applicable

Reviewer Expertise:

Pregnancy, maternity care, and perinatal mortality.

I confirm that I have read this submission and believe that I have an appropriate level of expertise to confirm that it is of an acceptable scientific standard, however I have significant reservations, as outlined above.

# Response

**Published:** 2024-03-21

**Authors:** Marie-Laure Volvert

**Affiliations:** King's College London, UK

Thank you for your generally positive comments and for your useful suggestions. We hope we have addressed these in the individual comments below and in the revised manuscript, where applicable.  

1. *A brief summary of PRECISE in the introduction*

  Thank you for this comment, we have included a bit more about PRECISE with relevant references included for further details about this study.  

1. *Under the Clinical data heading it states that women will be asked about both their and their child's health. How will they be asked? Will this be via survey or interview? How many language groups are included amongst the PRECISE-DYAD population and how will this be overcome?*

  We apologise that this was not totally clear and have tried to revise this to make these details clearer. All participants are being invited to participate, via phone calls and once signed consent has been obtained at their first visit, are invited back for up to 4 visits where different detailed clinical questions, neurodevelopmental and cardiological assessments and biological samples are collected at the various timepoints as detailed within the protocol manuscript.   

1. *The Mental Health study is referred to as a pilot study, but may be better described as a sub-study that will include a pilot phase.*

  We thank the reviewer for this suggestion and have now revised this accordingly as per the suggestion.  

1. *The quality of care sub-study would also benefit from detail on how data will be collected from women on their care experiences (survey/interview?).*

  Thank you for this, we have now clarified that this, each participant for this sub-study will have data collected via interviews at the appropriate visits.  

1. *The Air Quality sub-study will require participants (mothers presumably) to carry a device for 5 days. Further detail shroud be provided on the potential burden to participants and how this might be offset to ensure recruitment and retention.*

  These devices are not very heavy and fit in a small shoulder bag and following some community engagement it was found that mothers were happy to where these. Since the submission of this protocol, we have indeed completed this arm of the study and did not encounter any issues with burden it the participants.  

1. *Details on data collection instruments are also scant in the Health Economics sub-study.*

  Thank you for this. We have clarified that the data collection for the Health Economics sub-study has used measures of both family opportunity costs and economic resilience following pregnancy. *Finally, the study sample size consideration relates to placental disorders among the PRECISE-DYAD population and so it remains to be seen whether the group sizes of the substudy participants are sufficient in regard to the endpoints.*   Thank you for this point, these sub-studies are novel and will provide us with pilot data for feasibility and suggest any potential associations and thus an important part of the DYAD study. All data form these sub-studies, will have that caveat of limitation in numbers, but will provide value output for future studies, both with PRECISE-DYAD and for others. *There is some redundancy in the writing and further detail could be added on the specific methods for each sub-study without making the article excessively long.*   We hope in the revised manuscript with have added to those specific methodologies for the sub-studies and thus may the entire Protocol paper concise and suitable. *Despite the comments above, the group undertaking this work have already demonstrated the ability to carry out ambitious work in this context. Perinatal outcomes are considerably worse in sub-saharan Africa than in other parts of the world representing a greater burden for families and a downstream brake on social and economic advancement. Thus, there is an imperative to conduct research that may inform understandings of factors which promote healthy pregnancy and birth outcomes and those which contribute to harm. The PRECISE network have established the means to conduct such studies and have the skills and experience to do so, despite the challenges I have highlighted.*   We thank the reviewer for their positive words and are pleased to see that they understand the need for such studies and think that our ambitions for DYAD and beyond are of value and feasible

# Reviewer report: Reviewer response for version 1

**DOI:** 10.21956/wellcomeopenres.20478.r65530

**Published:** 2023-09-20

**Authors:** Tao Xue

**Affiliations:** 1 Peking University, Beijing, Beijing, China

In the manuscript, the authors describe the plan of the PRECISE-DYAD study in details. For me, the ongoing PRECISE-DYAD study is well designed and of public health importance in SSA. Before indexing, I encourage the authors to enhance their epidemiological design on sample selection, which should come first before their comprehensive data collection. Specifically, my comments are as follows.

1. The authors should mention some similar DYAD studies (completed or ongoing) in high-income countries as references for their readers. They are also encouraged to discuss what’s the novelty of the PRECISE-DYAD study, in comparison with those studies.
2. Now, the manuscript only briefly described their study domain. Since the planning study is nested within an extent cohort, PRECISE, the authors should have some data or summary statistics to describe the target population of the PRECISE-DYAD study quantitively.
3. Although the authors stated that they would use causal inference models to analyze their data, the PRECISE-DYAD might be failed to reveal causality due to the insufficiently-designed sample collection.
  1. First, the authors should clarify and specify their hypotheses. What are the major or secondary exposure of interests? What are the major outcomes of interests? Any biological evidences to generate a hypothesis for an exposure-outcome pair? Why DYAD design is required to test the hypothesis.
  2. Second, what are the efforts to make sure the randomization in the study?
  3. Third, all possible bias should be discussed in details. How will the PRECISE-DYAD study make efforts to minimize those biases.

Is the study design appropriate for the research question?

Yes

Is the rationale for, and objectives of, the study clearly described?

Yes

Are sufficient details of the methods provided to allow replication by others?

Yes

Are the datasets clearly presented in a useable and accessible format?

Yes

Reviewer Expertise:

Reproductive epidemiology; Environmental epidemiology.

I confirm that I have read this submission and believe that I have an appropriate level of expertise to confirm that it is of an acceptable scientific standard, however I have significant reservations, as outlined above.

# Response

**Published:** 2024-03-21

**Authors:** Marie-Laure Volvert

**Affiliations:** King's College London, UK

We thank the reviewer for taking time out to review our protocol paper and glad to see that they have approved it. Thank you for the constructive comments, which we have responded to point-by-point below and revised the manuscript accordingly:

1. *The authors should mention some similar DYAD studies (completed or ongoing) in high-income countries as references for their readers. They are also encouraged to discuss what’s the novelty of the PRECISE-DYAD study, in comparison with those studies.*

  Thank you for the comment, there are very few studies that have followed up mothers and their offspring DYADs up in such granular detail. There have been some similar studies completed on high-income countries (e.g. SCOPE), which we have now included as references in the introduction. The main novelty of this study is this is one of the first detailed pregnancy and child follow-up biorepositories covering East, West and Southern sub-Saharan Africa and includes both data as well as biological samples, alongside measures of child and neurological developmental measures. This has now been further emphasised in the revised manuscript.  

1. *Now, the manuscript only briefly described their study domain. Since the planning study is nested within an extent cohort, PRECISE, the authors should have some data or summary statistics to describe the target population of the PRECISE-DYAD study quantitively.*

  We have completed our initial round of data cleaning for the PRECISE inception cohort, and are on the point of submitting a manuscript that will describe the cohort and associated data and biological repositories. We will provide a reference to this paper in that submission to close the loop, and, once that paper is in press, upload the details onto the PRECISE network website.  

1. *Although the authors stated that they would use causal inference models to analyze their data, the PRECISE-DYAD might be failed to reveal causality due to the insufficiently-designed sample collection.*

1. *First, the authors should clarify and specify their hypotheses. What are the major or secondary exposure of interests? What are the major outcomes of interests? Any biological evidences to generate a hypothesis for an exposure-outcome pair? Why DYAD design is required to test the hypothesis.*

  Please see below our modified Aims section from the manuscript:   To test the overarching hypothesis that maternal-fetal-child health trajectories in sub-Saharan Africa are inextricably linked through social, clinical, and biomarker factors, the broad aim of PRECISE-DYAD is to provide an epidemiological and mechanistic understanding of maternal and infant health and development, in relation to *in utero* disease pathways in sub-Saharan African settings. The specific objectives are:  

1. To assess the effect of pregnancy complications on mothers (i) decisions around birth spacing (ii) subsequent pregnancy (iii) mental health (iv) child bonding (v) early-onset non-communicable disease (NCD) and multi-morbidities, and (vi) social functioning.  
2. To assess the measurable impact of pregnancy events on physical, mental, and neurodevelopmental health trajectories (specifically, moderate-to-severe neurodevelopmental, visual, or hearing disability) of children at 2-3 years of age.
3. To (i) identify the biological mechanisms that underpin relationships between intrauterine and early postnatal exposures, e.g. environmental or infectious diseases and maternal and child outcomes and (ii) investigate if health trajectories are modified by environmental exposures (e.g., air and water quality) and co-exposures (e.g., nutrition, lifestyle and quality of care) during the postnatal period and (iii) assess what the effects of caring for children with moderate-to-severe neurodevelopmental disability (vs those without such disability) are on: (a) subsequent pregnancy, (b) maternal mental health, and (c) early-onset NCDs and multi-morbidities.

 

1. *Second, what are the efforts to make sure the randomization in the study?*

  As this is an observational study, no randomisation is required. We have stated that these participants are already recruited through PRECISE and, therefore, randomisation is not applicable to this study. However, for some sub-studies (e.g., quality of care and health economics), we are randomly choosing cases and controls according to the presence or absence of a placental disorder in the index pregnancy.  

1. *Third, all possible bias should be discussed in details. How will the PRECISE-DYAD study make efforts to minimize those biases.*

  Thank you for your comment, from the previous PRECISE study, we have made every effort to collect as much as possible, a representative cohort of women and child DYADS and thus minimise bias.
